# Seasonal Mapping of Irrigated Winter Wheat Traits in Argentina with a Hybrid Retrieval Workflow Using Sentinel-2 Imagery

**DOI:** 10.3390/rs14184531

**Published:** 2022-09-10

**Authors:** Gabriel Caballero, Alejandro Pezzola, Cristina Winschel, Alejandra Casella, Paolo Sanchez Angonova, Juan Pablo Rivera-Caicedo, Katja Berger, Jochem Verrelst, Jesus Delegido

**Affiliations:** 1 Agri-Environmental Engineering, Technological University of Uruguay (UTEC), Av. Italia 6201, Montevideo 11500, Uruguay; 2 Image Processing Laboratory (IPL), University of Valencia, C/Catedrático José Beltrán 2, Paterna, 46980 Valencia, Spain; 3 Remote Sensing and SIG Laboratory, Hilario Ascasubi Agricultural Experimental Station, National Institute of Agricultural Technology (INTA), Hilario Ascasubi 8142, Argentina; 4 Permanent Observatory of Agro-Ecosystems, Climate and Water Institute-National Agricultural Research Centre (ICyA-CNIA), National Institute of Agricultural Technology (INTA), Nicolás Repetto s/n, Hurlingham, Buenos Aires 1686, Argentina; 5 Secretary of Research and Graduate Studies, CONACYT-UAN, Tepic 63155, Mexico; 6 Mantle Labs GmbH, Grünentorgasse 19/4, 1090 Vienna, Austria

**Keywords:** leaf area index, vegetation water and chlorophyll content, Gaussian processes regression, hybrid retrieval workflow, dimensionality reduction, active learning

## Abstract

Earth observation offers an unprecedented opportunity to monitor intensively cultivated areas providing key support to assess fertilizer needs and crop water uptake. Routinely, vegetation traits mapping can help farmers to monitor plant development along the crop’s phenological cycle, which is particularly relevant for irrigated agricultural areas. The high spatial and temporal resolution of the Sentinel-2 (S2) multispectral instrument leverages the possibility to estimate leaf area index (LAI), canopy chlorophyll content (CCC), and vegetation water content (VWC) from space. Therefore, our study presents a hybrid retrieval workflow combining a physically-based strategy with a machine learning regression algorithm, i.e., Gaussian processes regression, and an active learning technique to estimate LAI, CCC and VWC of irrigated winter wheat. The established hybrid models of the three traits were validated against in-situ data of a wheat campaign in the Bonaerense valley, South of the Buenos Aires Province, Argentina, in the year 2020. We obtained good to highly accurate validation results with LAI: R^2^ = 0.92, RMSE = 0.43 m^2^ m^−2^, CCC: R^2^ = 0.80, RMSE = 0.27 g m^−2^ and VWC: R^2^ = 0.75, RMSE = 416 g m^−2^. The retrieval models were also applied to a series of S2 images, producing time series along the seasonal cycle, which reflected the effects of fertilizer and irrigation on crop growth. The associated uncertainties along with the obtained maps underlined the robustness of the hybrid retrieval workflow. We conclude that processing S2 imagery with optimised hybrid models allows accurate space-based crop traits mapping over large irrigated areas and thus can support agricultural management decisions.

## 1 Introduction

Wheat is a worldwide cultivated grain crop providing nearly 20% of all calories consumed due to its strong adaptability to various temperature and water conditions [1]. As an important global food crop, wheat yield information is essential in terms of food security [2]. Good agronomic practices and natural resource usage become fundamental for winter wheat grain development and crop yield. These practices have an important significance for modern precision agriculture [3]. Irrigated valleys, although representing only 20% of the world’s croplands, produce 40% of the global crop harvest [4]. In arid and semi-arid intensively cultivated areas, irrigation improves economic returns and can boost food production by up to 400% [4,5]. Hence, accurate and timely mapping of winter wheat biophysical and biochemical variable, or crop traits, results in being indispensable for enhancing crop management, food security, and agriculture structure adjustment [6–8]. The estimation and monitoring of crucial crop variables, such as leaf area index (LAI), canopy chlorophyll content (CCC), and vegetation water content (VWC), are key for agricultural applications including crop growth modelling and yield estimation supporting sustainable management of cultivated areas [6,9]. Definitions of these variables are given as follows.

LAI is defined as the total one-sided leaf area per soil unit (m^2^ leaf area per m^2^ soil). It is strongly related to canopy photosynthesis and evapotranspiration and plays a key role in the exchange of energy and water between the biosphere and atmosphere [10]. Varying definitions of LAI have been presented, such as effective plant area index [11], which includes the area from all plant organs and assumes random distributions of leaves. The green LAI (GAI) is probably the most relevant term describing the radiation transfer in vegetated canopies, i.e., the green photosynthetically active elements of the canopy [12,13]. Here, we refer mainly to GAI being most closely related to the signal actually recorded by satellite instruments. Nonetheless, for the sake of simplicity, we use the term LAI throughout this study. CCC is defined as the product of LAI and leaf chlorophyll content(C_*ab*_) expressed in grams per unit leaf area (g/m^2^). Hence, CCC represents the optical path in the vegetated canopy where absorption by chlorophyll pigments dominates the radiometric signal [14]. In addition, due to its strong relationship with nitrogen (N) content, often C_*ab*_ is considered a proxy for leaf N status. Therefore, CCC can be considered for quantifying canopy-level N content [15,16], although restricted to vegetative growth stages [17]. Due to its strong association with plant transpiration, vegetation stress, and biomass productivity, VWC has been widely considered a crucial variable for crop physiological status [18–20]. VWC of wheat crops is also a significant growth indicator during different development stages [6]. Water availability not only affects wheat photosynthesis, but also the grain filling rate and, ultimately, yield [21,22]. Traditionally, wheat crop water content has been determined by manually sampling plants to obtain fresh weight and dry weight and then calculating crop water content [2]. However, conventional field measurements are destructive and labour-intensive, especially across large areas with significant within-field spatial variability in soil infiltration, drainage, and conductivity characteristics [23]. The same holds true for destructive sampling of other crop variables. Earth observation (EO) technology can be used as an appealing alternative, providing a synoptic view of the Earth’s surface by making use of the complex interactions between radiation and the environment [24]. Nowadays, an attractive operational EO system with the capability to capture the visible (VIS), near-infrared (NIR), red-edge and short-wave infrared (SWIR) spectral domains involves the Sentinel-2 (S2) mission [25]. The recent S2 constellation that combines a high revisit frequency (5-day) and spatial resolution (10–20 m) with systematic global acquisition and an open access policy is promising in the development of operational farming services in near-real-time [16]. In recent years, the rapid development of EO technology has produced an unprecedented amount of research applying satellite data for monitoring crop traits across large areas, in a quick and accurate way e.g., [13,19,26–30].

With respect to the retrieval of crop traits from EO data, a multitude of techniques have been developed, which can be categorized into four main categories [30,31]: (1) Parametric regression approaches typically consist of relations between the traits and spectral data transformed into spectral indices e.g., [32,33]. (2) The second category of regression approaches obtains these variables from reflectance data using linear or nonlinear nonparametric approaches, i.e., chemometrics or machine learning regression algorithms (MLRA) e.g., [34–36]. (3) The category of physically-based retrieval methods refers to the inversion of radiative transfer models (RTMs) e.g., [37–41]. RTMs apply physical laws to explain the cause–effect relationships between radiation–photon interactions and plant constituents. The most prominent one-dimensional, (1D) turbid medium RTM for sim-ulating vegetation reflectance is PROSAIL [42]. PROSAIL is composed of a leaf optical properties model, typically from the PROSPECT family [43,44], and, (b) the Scattering of Arbitrarily Inclined Leaves canopy architecture model (SAIL, 4SAIL) [45,46]. The last category of retrieval methods (4) is presented by hybrid approaches, being perhaps of most interest within current research lines and also for operational contexts [26,27,30,47,48]. Hybrid methods blend physics described by RTMs with the speed and efficiency of MLRAs. Within hybrid scenarios, training datasets are simulated by varying the input parameter space of coupled leaf-canopy RTMs. The nonlinear relationships between simulated reflectance and vegetation properties are then learned by an MLRA. Ideally, these training databases represent the canopy states realistically and at the same time are small enough to avoid exhaustive processing times. Regarding these strategies, a diversity of MLRAs have been successfully applied for retrieval tasks during the last few decades, e.g., artificial neural networks, decision trees or kernel-based methods [30,31]. As part of the latter family, especially Gaussian processes, regression (GPR) [49] emerged as a competitive retrieval algorithm e.g., [50–52], and has been widely adopted in studies inferring traits from EO data e.g., [48,53–64].

GPR received special attention also thanks to associated uncertainty estimates provided along with the predictions. This special feature enables assessing the fidelity of the models when transferring them into other spaces and times [28]. Moreover, to enhance mapping performance and processing speed, active learning (AL) techniques can be incorporated into hybrid workflows. AL aims to optimize training datasets through intelligent sampling using an iterative procedure devoted to exploiting samples during the design of the regression model [65–67].

Time-series of multiple quantitative traits over irrigated winter wheat present a captivating tool to address crop management questions and improve agricultural practises. Multiple authors have developed satellite-based retrieval models for wheat traits mapping using MLRA or parametric regressions [3,13,20,59,68,69]. Hybrid retrieval workflows may outperform these simpler empirical approaches due to their appealing property of combining physical awareness (RTMs) with flexibility of machine learning algorithms. Hence, they are an appealing alternative to be explored in our context. Several experimental studies have already demonstrated the feasibility of producing accurate vegetation traits maps from multispectral data, including S2 [59,70–73]. However, there is still the need to further test and adapt hybrid methods under diverse environmental conditions, at different locations and to multiple crop types.

In an attempt to demonstrate the possibilities of space-based cropland trait monitoring, this study presents the development of an optimised hybrid retrieval workflow dedicated to irrigated winter wheat monitoring in Argentina. Given the above-sketched general framework, this study aims to reach the following objectives: (1) to develop independent hybrid models optimised with AL and GPR for an explicit quantification of winter wheat LAI, CCC, and VWC from S2 data; (2) to generate accurate S2-based maps of the wheat traits with the inclusion of associated uncertainties over an intensive irrigated agroecosystem; and (3) to evaluate LAI, CCC, and VWC time series identifying seasonal trends over the selected study site.

## 2 Materials and Methods

### 2.1 Generation of Training Data Sets

Aiming to develop optimised hybrid models, the workflow initiates with simulating a training database using the coupled PROSPECT-PRO [44] and 4SAIL models, further referred to as PROSAIL-PRO. The models simulate bi-directional reflectance as a function of diverse leaf biochemical input variables, e.g., C_*ab*_, leaf protein content (C_*p*_), leaf carotenoid content (C_*xc*_) or leaf equivalent water thickness (EWT) and biophysical variables, i.e., LAI and average leaf inclination angle (ALIA). The synthetic training database was generated by using Latin Hypercube Sampling (LHS) to randomly select samples from the multi-spectral input space. For this, 1000 combinations were drawn from all PROSAIL-PRO parameters. The selected size of the training dataset may appear rather small compared to classical retrieval approaches, for instance using lookup-table approaches or neural networks. Note that a standard implementation of GPR struggles to cope with thousands of samples within reasonable time intervals. Hence, as suggested by prior studies, e.g., [67,74], successfully training GP models with data sets of rather small sizes, we also restrict here to 1000 samples.

An overview of all included PROSAIL-PRO parameters including sampling, mean values, distributions and ranges is given in Table 1 (see also the study by Berger et al. [26] for full information about the generation of the training database). VWC can then be directly calculated from EWT with a (unit) conversion factor of 10’000 upscaling from leaf to canopy level through LAI. Fractional vegetation cover (FVC) was used to introduce the crop-bare-soil relationship (see Equation (1)). CCC was also obtained through extrapolating from C_*ab*_ to canopy level by means of LAI (see Equation (2)). Finally, simulated VWC and CCC both in [g m^−2^] were added to the training database:

(1) $$VWC_{s}=(EWT\times LAI\times FVC)\times 10000\left[\text{g}/{\text{m}}^{2}\right]$$

(2) $$CCC_{s}=\left(C_{ab}\times LAI\right)/100\left[\text{g}/{\text{m}}^{2}\right]$$

Finally, bi-directional canopy reflectance was calculated using S2 spectral configuration and excluding low-resolution bands of 60 m since their focus is on cloud screening and atmospheric corrections. For instance, Estévez et al. [59] analyzed the contribution of the S2 spectral bands covering 10–20 m pixel resolutions for LAI retrieval purposes. The authors concluded to keep all ten bands (with S2 central wavelengths of 493 nm, 560 nm, 665 nm, 704 nm, 740 nm, 783 nm, 833 nm, 865 nm, 1610 nm, and 2190 nm) for further processing. To assure a maximum of spectral information required for the retrieval of multiple crop traits, we also decided to explore the ten S2 bands.

### 2.2 Gaussian Processes Regression

GPR is used as core MLRA in the hybrid model development. GPR modeling is flexible enough to fit many types of data, including geospatial and time-series data. GP is a Gaussian distribution over functions, which means that, instead of inferring the distribution of a parameter, GP can be then used to infer a distribution over functions directly under the premise that the function values are themselves random variables. At the inference stage, every time a new observation is made, the model hypothesis (prior probability distribution) is updated in light of the new observations. After having observed some function values, prior distribution over functions can be converted into posterior over functions. A Gaussian Process is a collection of random variables any finite number of which have (consistent) joint Gaussian distributions [49].

Notationally, GP can be deployed as follows: we observe training dataset 𝔇 = {(*x_i_, f_i_* (*x_i_*))|*i* = 1, …, *N*}, where *x_i_* ∈ ℝ^ℬ^ (being ℬ the number of sensor spectral bands) and $X={\left\{x_{i}\right\}}_{i=1}^{N}$, GP aims to provide a suitable model to predict the function outputs f_*_ given a test dataset X_*_ of size N_*_ × 𝔇 unknown by the model. In a noise-free environment, the joint distribution under GP is:

(3) $$\left(\begin{array}{c} f\\ f_{*} \end{array}\right)∼𝒩\left(\left[\begin{array}{c} \mu \\ \mu_{*} \end{array}\right],\left[\begin{array}{cc} k(X,X) & k\left(X^{,}X_{*}\right)\\ k\left(X_{*},X\right) & k\left(X_{*},X_{*}\right) \end{array}\right]\right)$$

where *μ* and μ_*_ are the means of the functions *f* and f_*_, which can be assumed as 0, *K* = *k*(*X*, *X*) is the matrix of self-similarities in the training dataset, K_*_ = *k*(X, X_*_) and $K_{*}^{T}=k\left(X_{*},X\right)$ represents the similarities between training and test datasets, and K_**_ = *k*(X_*_, X_*_) express the self-similarities in the test dataset. The mean μ_*_ (expectation of f_*_) and the co-variance ∑_*_ of the test dataset X_*_ can be calculated as follows:

(4) $$\mu_{*}=\text{𝔼}\left(f_{*}\right)=K_{*}^{T}K^{-1}f$$

(5) $${\text{Σ}}_{*}=K_{**}-K_{*}^{T}K^{-1}K_{*}$$

To compute the measures of similarity between two points *x_i_* and *x_j_*, a positive definite kernel (or covariance function) that describes the covariance of the GP random variables should be implemented. The squared exponential kernel, also known as a radial basis function (RBF), which arises from taking the exponent of the scaled squared Euclidean distance between the data locations, is one of the most popular kernels used in GP modeling. It can be computed as:

(6) $$k\left(x_{i},x_{j}\right)=\sigma_{f}^{2}e^{-\frac{1}{2\ell^{2}}\left\|x_{i}-x_{j}{}^{2}\right\|}$$

where $\sigma_{f}^{2}$ is the overall variance ($\sigma_{f}^{2}$ that is also known as amplitude), and the parameter 𝓁 is the variance of the Gaussians (length scale) that controls the smoothness and confidence of the regression process. Thus, a Gaussian process is a distribution over functions whose smoothness is defined by the kernel *k*. RBF is a linear combination of basis functions; wherever there is data, we place a basis function scaled by the term $\sigma_{f}^{2}$. Consequently, f_*_ is the sum of basis functions scaled by $\sigma_{f}^{2}$. There are several manners to find the optimal 𝓁 value, cross-validation, maximum likelihood estimation (MLE), and Bayesian Learning are examples of different methods that can be implemented in the stage of learning the kernel parameters.

The key concept regarding GP is the fact that given two points *x_i_* and *x_j_* that are similar (similarity determined by the kernel k), the function values *f* (*x_i_*) and *f* (*x_j_*), should be expected to be similar too. Straight away, the kernel function parameters have been optimised using a training dataset, and the kernel matrix can be plugged into the predictive equations for the mean μ_*_ and co-variance Σ_*_ of the test data x_*_ to obtain predictions f_*_ on the whole test dataset X_*_. The process of making predictions is only computing the posterior distribution *p*(f_*_|X_*_, *X, f*) of x_*_ given *f* and the previous *x*, we can condition on the observations, to obtain:

(7) $$f_{*}|X_{*},X,f∼𝒩\left(f_{*};k\left(X,X_{*}\right)k{\left(X,X\right)}^{-1}f,k\left(X_{*},X_{*}\right)-k\left(X,X_{*}\right)k{\left(X,X\right)}^{-1}k\left(X_{*},X\right)\right)$$

Conditioning a Gaussian gives another Gaussian:

(8) $$p\left(f_{*}|X_{*},X,f\right)∼𝒩\left(f_{*}|\mu_{*,}\sum^{\text{​}}*\right)$$

where Σ_*_ models the co-variances and cross-covariances between all combinations of train and test data. Predictions made using GP are not just point predictions: they are whole probability distributions.

More commonly, we have access to noisy observations ${\left\{x_{i},y_{i}\right\}}_{i=1}^{N}$ with *y_i_* = *f_i_*(*x_i_*) + ***ε***, where ***ε*** is the noise of the observations. Assuming that the noise is Gaussian distributed with zero mean and variance $\sigma_{n}^{2}$ the noise can be therefore expressed as $\varepsilon ∼𝒩\left(0,\sigma_{n}^{2}\right)$. The value of $\sigma_{n}^{2}$ can be estimated from data utilizing the MLE principle. Assuming independently added Gaussian noise to each observation, the joint distribution of observations and test predictions is given by:

(9) $$\left(\begin{array}{c} y\\ f_{*} \end{array}\right)∼𝒩\left([0],\left[\begin{array}{cc} k(X,X)+\sigma_{n}^{2}I & k\left(X,X_{*}\right)\\ k\left(X_{*},X\right) & k\left(X_{*},X_{*}\right) \end{array}\right]\right)$$

To make predictions from a noisy GP, the only thing to do is to establish a conditioning on the observation:

(10) $$f_{*}|X_{*},X,y∼𝒩\left(k\left(X,X_{*}\right){\left[k(X,X)+\sigma_{n}^{2}I\right]}^{-1}y,k\left(X_{*},X_{*}\right)-k\left(X,X_{*}\right){\left[k(X,X)+\sigma_{n}^{2}I\right]}^{-1}k\left(X_{*},X\right)\right)$$

The mean is linear in two ways:

(11) $$\mu_{*}=k\left(X_{*},X\right){\left[k(X,X)+\sigma_{n}^{2}\right]}^{-1}y$$

The predictive co-variance (confidence intervals) is the difference between two terms:

(12) $${\text{Σ}}_{*}=k\left(X_{*},X_{*}\right)-k\left(X_{*},X\right){\left[k(X,X)+\sigma_{n}^{2}I\right]}^{-1}k\left(X,X_{*}\right)$$

The first term is the prior variance, from which we subtract a (positive) term, telling how much the data *X* has explained. It is worth mentioning that the variance is independent of the observed outputs *y*. Although the posterior distribution *p*(f_*_|X_*_, *X, y*) covers noise in training data, it is still a distribution over noise-free predictions f_*_. To additionally include noise *ε* into predictions y_*_, we must add $\sigma_{n}^{2}$ to the diagonal of Σ_*_:

(13) $$p\left(y_{*}|X_{*},X,y\right)∼𝒩\left(y_{*}|\mu_{*},\sum^{\text{​}}*+\sigma_{n}^{2}I\right)$$

Summarizing, as a Bayesian approach, GPR provides a natural and automatic mechanism to construct and calibrate uncertainties.

### 2.3 Active Learning Principles

GPR as a kernel-based regression method permits an enlarged number of input variables to be scanned and can be particularly convenient to deal with regression uncertainties since the confidence interval for the predictions is provided. Even though GPR offer a suitable alternative to processing large training datasets, processing large simulated training datasets could become computationally inefficient. Consequently, a reduction in the sampling domain, which specifies the size of the training dataset, is needed. A solution to the sampling reduction issue is given by semi-supervised approaches, and these techniques are also known as active learning [66]. AL aims to provide a strategy to efficiently reduce massive training datasets into an optimal dataset by sampling and evaluating the data pool iteratively through an intelligent process [67]. In AL, new informative samples are labeled based on the knowledge acquired during the exploration of the data input space.

Solving regression problems with AL can be addressed through a diversity framework. Diversity-based AL strategies create a reduced training dataset by selecting new samples from the input space, according to their dissimilarities [75]. Euclidean distance-based diversity (EBD) selects those samples out of the pool being distant from the already included ones in the training set, using squared Euclidean distance:

(14) $$d_{E}={\left\|x_{u}-x_{l}\right\|}_{2}^{2}$$

where *x_u_* is a sample from the candidate set, and *x_l_* is a sample from the training dataset. All distances between samples are computed and then the farthest are selected.

With the purpose to decrease the GPR training time, the EBD sampling method was performed in order to reduce the size of the input data space. Accordingly, 1% of the 1000 labeled samples (pairs of simulated bi-directional reflectance and vegetation traits) was randomly selected as the initial data set. The GPR training process was iteratively repeated up to 1000 times. At each iteration, the sample from the input data set with the largest distance between the pool of the 1000 labeled samples was selected by the EBD and added to the training data only when performance improved as evaluated by the root mean square error (RMSE) against the in-situ data [66,72].

### 2.4 Study Site

**Figure 1:**
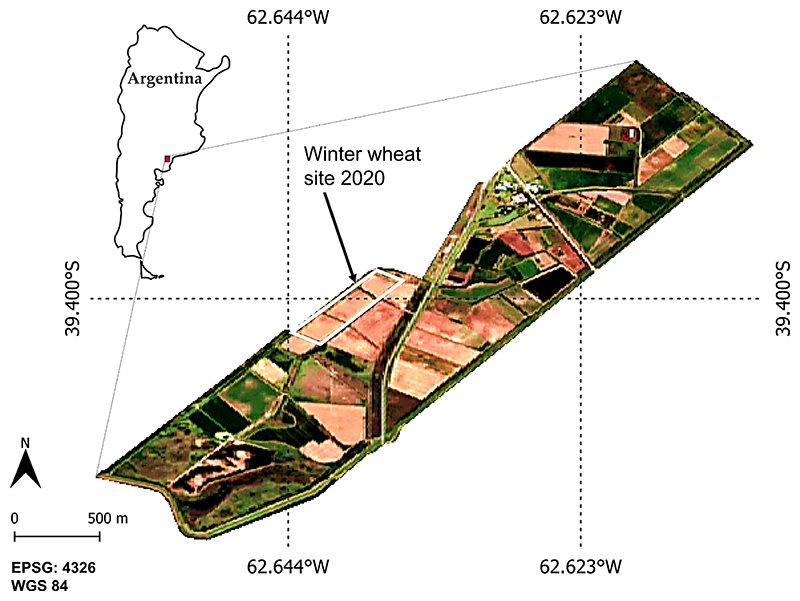
Overview of the Bonaerense Valley of Colorado River study site with test fields for the winter wheat campaign of the year 2020. True colour S2 image (R = B4, G = B3, B = B2) of 27 December 2020. Reference system: WGS84 (EPSG 4326).

The Bonaerense Valley of Colorado River (BVCR) is located in the South of the Buenos Aires Province, Argentina, between the 39° and 40°S parallels and the 62° and 63°W meridians. Two different cropping systems can be well distinguished in the region: irrigated and non-irrigated crops. From the productive point of view, irrigation presents the highest productivity and contribution to the regional socio-economic movement. The area has a surface of 500,000 ha, of which 140,000 ha are irrigated by an extensive irrigation network based on uncoated drainage channels. Gravity irrigation has made the most of the agricultural activities in the area possible. The implanted crops under irrigation conditions occupy 91163 ha in the BVCR, including horticulture, pastures, and cereals. This study focused on three wheat paddocks of the BVCR in the Villarino district (see Figure 1).

### 2.5 Wheat Crop Experimental Design

A total of three repetitions were set up for each of the wheat paddocks. The position of nine elementary sampling units (ESU) was measured with a differential global positioning system (GPS) and recorded as point sample references in the field campaign database. The ESUs were revisited at each date corresponding to cloud-free S2 acquisitions to guarantee solid time-series. If there was a coincidence in ±6 days related to the S2 acquisition, the traits of the wheat crop were sampled.

**Figure 2:**
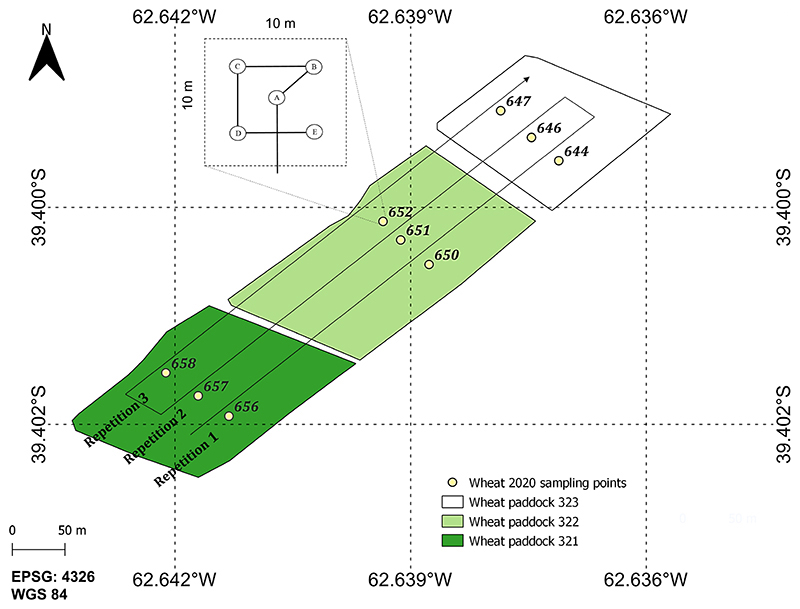
Layout of measurement design for the 2020 campaign at the BVCR study site: three ESUs were defined per wheat paddock and sampling approach for each elementary sampling unit, partly adapted from [69]. Reference system: WGS84 (EPSG 4326).

The measurements were performed following a defined field protocol for the BVCR 2020 wheat campaign; see Figure 2. Hereby, the sampling strategy consisted of defining ESUs of 10 m ×10 m size for each wheat paddock in order to comply with the S2 spatial resolution (10 m). In addition, a minimum distance of 30 m from the parcels’ edges was kept. Sampling points were distributed within the ESUs following a five-measurements square spatial sampling strategy providing a statistically averaged LAI estimate per ESU [69]. The centre of the ESUs (sampling point A) was georeferenced using the S2 pixel grid to assure correspondence to the S2 reflectance data.

The in-situ measurement protocol used to carry out the field campaigns involved the collection of four main traits: LAI, fractional vegetation cover (FVC), C_*ab*_, and above-ground fresh biomass (AGFB). The crop phenological stage and field data observations were also registered in the in-situ measurements database. The phenology was determined according to secondary growth stages of the Zadoks-scale [76].

#### Wheat Crop Management and Field Data Collection

The wheat crop was sown on 25 June 2020 at three different paddocks in the study site, a uniform seed density was established at 95 kg ha^−1^, and the weight of 1000 seeds was 45.8 g on average. At the wheat sowed stage, 80 kg ha^−1^ of diammonium phosphate was used as a fertilizer considering the formulation 18-46-00 (18% N, 46% P_2_O_5_, and K_2_O 0%). Phosphorus fertilization is a key factor in replenishing soil nutrients and obtaining more vigorous plants. Moreover, a rapid formation and growth of the root system are promoted making plants more resistant to water deficit. A system of ground edges separated by 14 m was implemented at the pre-sowing labour stage to ensure water embankments during the irrigation processes. Sunflower hybrid seed was the predecessor crop. At the beginning of the tillering stage, a value of 243 wheat plants per square meter (plant density) was measured. The distance between rows (rows spacing) was set as 17.5 cm according to wheat crop management recommendations in the region of the Colorado river valley. Wheat fertilization took place in two instances throughout the phenological cycle, first on 31 August 2020 and second by the middle of September. The dose of nitrogen (Urea) supplied was distributed uniformly and ranged between 150–200 kg ha^−1^. It is reasonable to expect that wheat plants start to receive nutrients from Urea up to one week later of fertilization. Three gravity irrigations were performed at different instances of the crop cycle between late August and November.

A collection of field data was obtained from the study area of the BVCR. In the year 2020, the test site was visited regularly during the growing periods from August to December. Data were collected on wheat fields belonging to the communal farmlands of BVCR by a group of expert professionals and technicians of HAEE-INTA, Argentina. The exact sampling sites were the same across the wheat 2020 field campaign. Each site was confined to a 10 ×10 m area, corresponding to the average S2 pixel size.

LAI measurements were taken using the PocketLAI R Smart-App [77]. Six observations were made-up per ESU and averaged giving a total of 54 LAI values for the entire campaign.

The FVC was measured utilizing the “Canopeo®” App [78]. The app uses the RGB camera of the smart devices applying the relations R/G, B/G, and 2G-R-B to determine the crop canopy coverage percentage related to the soil.

To determine the AGFB value, five wheat plants per ESU were cut at soil level at each sampling date throughout the growing season. A sowing track or an area of 0.02 m^2^ was considered to obtain the AGFB samples. Each fresh biomass sample made up of wheat leaf, stalk, and fruit was enclosed in a sealed plastic bag and brought to the laboratory inside of a cooler for the fresh weight (FW) and dry weight (DW) measurements (see Table 2). The AGFB samples were weighed in the fresh state and oven-dried until reached constant weight for 24 h at 60 °C before dry weight was determined [79]. The DW corresponds to the above-ground dry biomass (AGDB) vegetation biophysical variable expressed in g units.

The SPAD-502 instrument (Minolta r) was used to perform C_*ab*_ measurements. Six leaves per wheat plant were selected randomly at each ESU, and five SPAD values were registered per wheat leaf and then averaged giving a total of 54 C_*ab*_ values. To extrapolate from SPAD non-dimensional values to C_*ab*_ values expressed in μg cm^−2^, the following Equation (15) was implemented:

(15) $$C_{ab}\left[\frac{\mu \text{g}}{{\text{cm}}^{2}}\right]=12.23e^{0.0279.SPAD}$$

LAI was used to upscale from C_*ab*_ to CCC. Factor 100 led to the correct ground surface unit by upscaling from leaf [μg cm^−2^] to canopy level [g m^−2^]:

(16) $$CCC\left[\frac{\text{g}}{{\text{m}}^{2}}\right]=C_{ab}\left[\frac{\mu \text{g}}{{\text{cm}}^{2}}\right]\times LAI\times \frac{1}{100}$$

Plant leaf water content is commonly expressed as equivalent water thickness corresponding to a hypothetical thickness of a single layer of water averaged over the whole leaf area [80]. EWT relates the leaf’s water content to the leaf’s area (Al), so it is usually expressed in μg cm^−2^ as follows:

(17) $$EWT=\frac{\left(FW-DW\right)}{Al}\left[\mu {\text{g}\;\text{cm}}^{-2}\right]$$

Canopy water content (CWC) is commonly derived through extrapolation by means of the LAI:

(18) $$CWC=EWT\times LAI\left[{\text{g}\;\text{m}}^{-2}\right]$$

Due to the linkage of LAI to the area of leaves, CWC may neglect the water content contained in other organs, such as stalks and fruits. To monitor the total amounts of water stored in wheat plants, leaves, stalks, and fruits were included in our analysis. The FW and DW values determined in the laboratory per each ESU of the study site (consisting of wheat fresh and dry organic matter) were used as a proxy for the determination of VWC expressed in g m^−2^ units. The VWC values were then obtained by calculating the difference between the FW [g] and DW [g] and referring it to the sowing area A, implicated in the field data collection process:

(19) $$VWC_{h}=\frac{\left(FW-DW\right)}{A}\left[{\text{g}\;\text{m}}^{-2}\right]$$

Equation (19) assumes a wheat plant homogeneously distributed in one square meter of soil, hence we refer here to *VWC_h_*, with the subscript *h* standing for homogeneous. However, this approach can lead to errors in the estimation of total VWC values. During the first growing stages, plants are separated by rows leading to a proportion of the soil visible to the sensor. Since the S2 sensor acquires surface reflectance in a 10 m × 10m ground sampling distance (GSD), the spectral value of each band summarizes both the soil and vegetation contributions. Only when the wheat crops reach the maximum greenness stage is the soil completely covered by vegetation. Hence, to be more precise in the determination of the VWC, we introduced a modification considering in-situ measured FVC in the calculation of plant water content per square meter of sowed wheat:

(20) $$VWC=VWC_{h}\times FVC=\frac{\left(FW-DW\right)}{A}\times FVC\left[{\text{g}\;\text{m}}^{-2}\right]$$

The calculated wheat CCC and VWC values were recorded as new inputs of the field campaign database as shown in Table 3.

**Figure 3:**
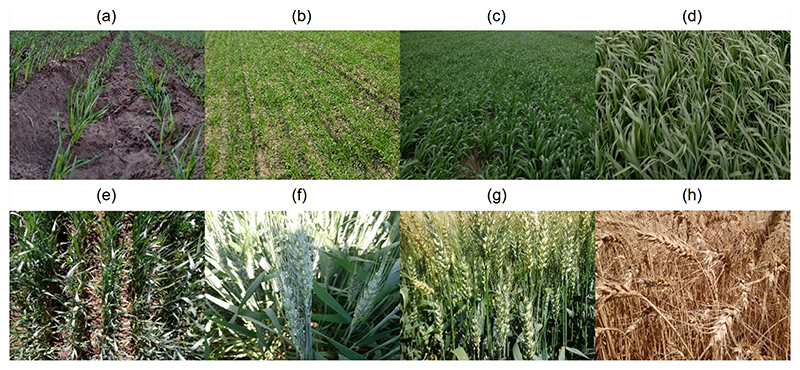
Photographic documentation of wheat crop growing period from August to November 2020, BVCR campaign, corresponding to LAI, FVC, *C_ab_*, and AGFB sampling dates. With (**a**) seedling stage at 10 August 2020; (**b**) tillering stage at 4 September 2020; (**c**) tillering stage at 17 September 2020; (**d**) tillering stage at 2 Ocotber 2020; (**e**) ear emergence from boot at 19 October 2020; (**f**) anthesis stage at 2 November 2020; (**g**) dough development at 30 November 2020, first appearance of senescence; (**h**) ripening stage at 16 December 2020, complete senescence.

The wheat phenological stage was registered along the BVCR campaign from August to December 2020 (see Figure 3 and Table 4).

### 2.6 Sentinel-2 Image Acquisitions

A series of S2 images consisting of 15 cloud-free L1C products from 29 August to 11 January 2020 was downloaded from ESA’s web server https://scihub.copernicus.eu/ (accessed on 20 June 2022).The S2 images were atmospherically corrected using the Sen2cor plugin [81] to obtain bottom-of-atmosphere (BOA) reflectance data from top-of-atmosphere (TOA) L1C products. As a result, 15 level 2A (L2A) S2 images were obtained, coinciding with ±6 days of in-situ sampling. The images were resampled to 10 m GSD and cropped according to the study site in the SNAP 7.0 software https://step.esa.int/main/snap-7-0-released/ (accessed on 18 July 2022).Bands B1 (443 nm), B9 (940 nm), and B10 (1375 nm) were excluded from the resulting products due to their low GSD of 60 m, being in correspondence to the simulations. Finally, the S2 products composed of ten spectral bands were used for further analysis within the AL optimization process (see Table 5).

### 2.7 Delineation of the Hybrid Retrieval Workflow

**Figure 4:**
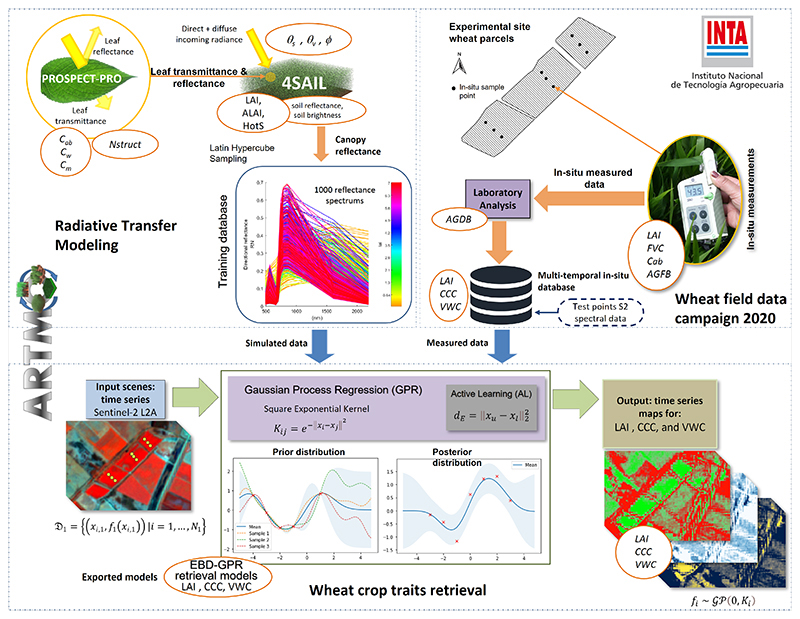
Illustration of the hybrid retrieval workflow using the coupled PROSAIL-PRO models to establish a training database for the GPR, partly adapted from [82]. The output maps show our vegetation traits modeling over the BVCR area in Argentina.

An overview of the complete hybrid retrieval workflow is given in Figure 4. Three well- distinguished conceptual blocks are detailed, starting with an RTM section, followed by in-situ data collection, and the retrieval of biochemical and biophysical traits. In summary, the implemented retrieval workflow consisted of the following four main steps:

1. generation of the training database, i.e., simulated TOC reflectance with corresponding traits using the PROSAIL-PRO model;
2. building the in-situ database containing multitemporal field measurements from the BVCR site and S2 spectra;
3. optimizing the training database with AL-EBD and GPR, applying retrieval models to obtain wheat LAI, CCC, and VWC; and
4. seasonal mapping of the three crop traits over irrigated wheat fields and corresponding uncertainties using S2 scenes.

Note that, according to the 2020 wheat campaign observations (see Table 4), the senescence of the crops started on 16 November 2020. In order to prioritize the retrieval models’ response within the vegetation greenness period, the in-situ database was restricted from 3 September to 16 November 2020. Additionally, 12 non-vegetated and 27 senescent crop samples were added to the database aiming to improve the robustness of the crop traits retrieval models after starting yellowing and senescing crop growth stages (see Appendix A for more information on the training database). For model evaluation, the coefficient of determination (R^2^), the RMSE, the normalized root mean square error (NRMSE), the mean absolute error (MAE), and the mean absolute percentage error (MAPE) were registered for each crop trait model.

The hybrid workflow was entirely built within the Automated Radiative Transfer Models Operator (ARTMO) toolbox [83]. ARTMO was developed as a modular graphical user interface in Matlab, to automate the simulation of RTMs [84]. The toolbox brings multiple RTMs together with essential tools required for the retrieval of a diversity of biophysical and biochemical vegetation traits. ARTMO has been expanded over the years with all kinds of RTMs and image processing options, such as the MLRA toolbox [85] with included AL module [74], emulation, sensitivity analysis, and scene generation. More information can be found at: http://artmotoolbox.com/ (accessed on 18 July 2022).

## 3 Results

### 3.1 Optimized Sample Selection for LAI, CCC and VWC Modeling

**Figure 5:**
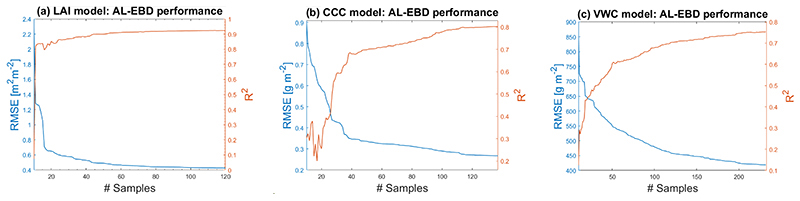
Goodness-of-fit results (RMSE, R^2^) using AL (EBD) against validation data. (**a**) LAI model; (**b**) CCC model; (**c**) VWC model.

In order to build efficient GPR-based retrieval models for wheat traits, at first, the AL EBD technique was explored for optimizing the training samples. In Figure 5, we demonstrate the results of training database reduction through EBD as a function of RMSE (left *y*-axis) and R^2^ (right *y*-axis). For all three variables, an initial dataset of 10 samples was used as starting point for the AL procedure.

Figure 5a shows the effect of the training data size on the LAI models’ accuracy, with optimal results obtained after adding 112 samples (RMSE: 0.42 m^2^ m^−2^; R^2^: 0.92). For CCC, the highest accuracy was obtained after adding 137 samples reaching RMSE: 0.27 g m^−2^ and R^2^: 0.8 (see Figure 5b). Figure 5c, shows that the optimal accuracy of the VWC model was achieved after adding 232 samples (RMSE: 416 g m^−2^; R^2^: 0.76). Note hereby that lowering the RMSE does not necessarily go along with an improvement of R^2^, as can be read on the right *y*-axis of Figure 5. Although it follows the same general trends as RMSE, the pattern provided by R^2^ is more irregular, indicating it as a less reliable measure than RMSE for AL testing [72].

**Figure 6:**
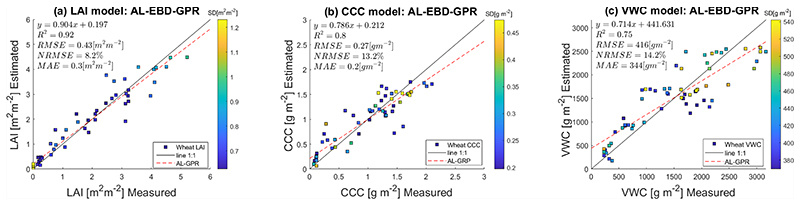
Measuredvs. estimated wheat traits along 1:1-line including uncertainty intervals, using the EBD-optimised training data set. (**a**) LAI model estimates; (**b**) CCC model estimates; (**c**) VWC model estimates.

Figure 6 presents the scatter plots of estimated against in-situ measured LAI, CCC and VWC samples. Overall, values for R^2^ and RMSE indicate relatively high agreements between modelled and measured wheat traits. In addition, uncertainties are provided as standard deviation (SD) expressed by the colour table. The SD of LAI, ranging from 0.62 to 1.23 m^2^ m^−2^, provides a proxy of the model’s uncertainty and is indicated by the colour bar close to the regression graph (see Figure 6a). Figure 6b shows the resulting optimised CCC model, with uncertainty interval ranging from 0.2 to 0.48 g m^−2^. In Figure 6c, the optimised VWC model are displayed, with uncertainty interval ranging from 370 to 542 g m^−2^.

### 3.2 Lai, CCC, and VWC Mapping

Next, the optimally trained hybrid GPR models were applied to the six selected dates of the 2020 BVCR wheat campaign. The scenes cover a variety of crop types, including the wheat paddocks selected for this study. Non-vegetated land covers, comprising bare soils, water bodies, and man-made surfaces, were removed from the scenes by applying a vectorial mask.

**Figure 7:**
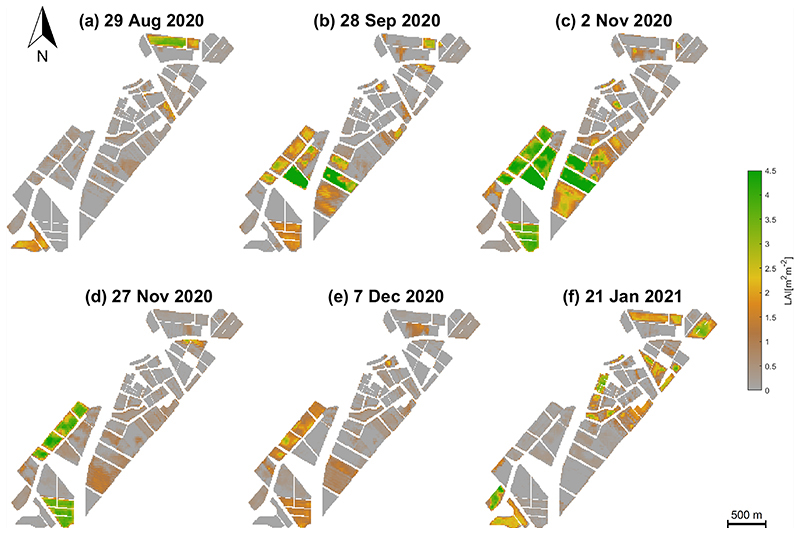
LAI [m^2^ m^−2^] of different crops in the study site, retrieved by the GPR model using simulated vegetation reflectance spectra, in-situ traits measurements and S2 surface multi-spectral reflectance data. Data obtained from BVCR wheat campaign 2020. (**a**) wheat tillering stage at 29 August 2020; (**b**) wheat booting stage at 28 September 2020; (**c**) wheat anthesis-flowering stage at 2 November 2020; (**d**) wheat dough development stage at 27 November 2020; (**e**) wheat ripening stage at 7 December 2020; (**f**) harvested wheat at 21 January 2021.

**Figure 8:**
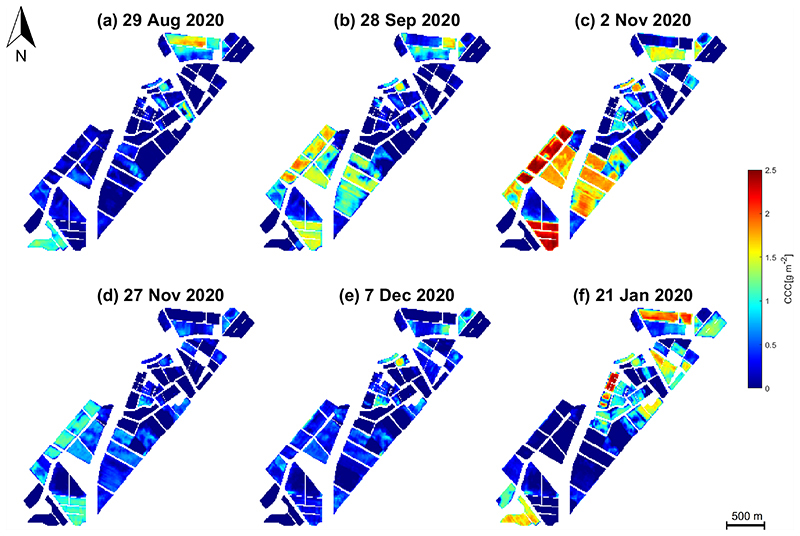
CCC[g m^−2^] of different crops in the study site, retrieved by the GPR model using simulated vegetation reflectance spectra, in-situ biophysical/biochemical measurements and S2 surface multi-spectral reflectance data. Data obtained from BVCR wheat campaign 2020. (**a**) wheat tillering stage at 29 August 2020; (**b**) wheat booting stage at 28 September 2020; (**c**) wheat anthesis-flowering stage at 2 November 2020; (**d**) wheat dough development stage at 27 November 2020; (**e**) wheat ripening stage at 7 December 2020; (**f**) harvested wheat at 21 January 2021.

Figure 7 displays the six S2-derived LAI maps over the BVCR site during the growing season of 2020 (ending 01/2021). All grey-coloured areas correspond to fallow or harvested fields or dried-out natural vegetation. In contrast, LAI distribution of the intensively cultivated crops may vary during the whole growing cycles of the BVCR 2020 campaign. The wheat crop was sown in late June 2020, leading to the emergence of the main wheat stem and three tillers in late August. The highest LAI values during the growing season are identified: LAI reaches a maximum greenness stage at the beginning of November 2020 with 4.12 m^2^ m^−2^. Wheat senescence starts in the middle of November and harvest takes place by the first days of January 2021. Figure 8 shows the multitemporal maps resulting from the CCC estimates for the BVCR study site. The CCC values oscillate between 0 and 2 g m^−2^ being the maximum detected on 2 November. Later in the growing season, from around 16 November 2020 onwards, all wheat parcels exhibited a markedly low CCC driving toward senescence. Due to the linkage between LAI and CCC, both variables are strongly correlated across the whole wheat’s growing cycle.

**Figure 9:**
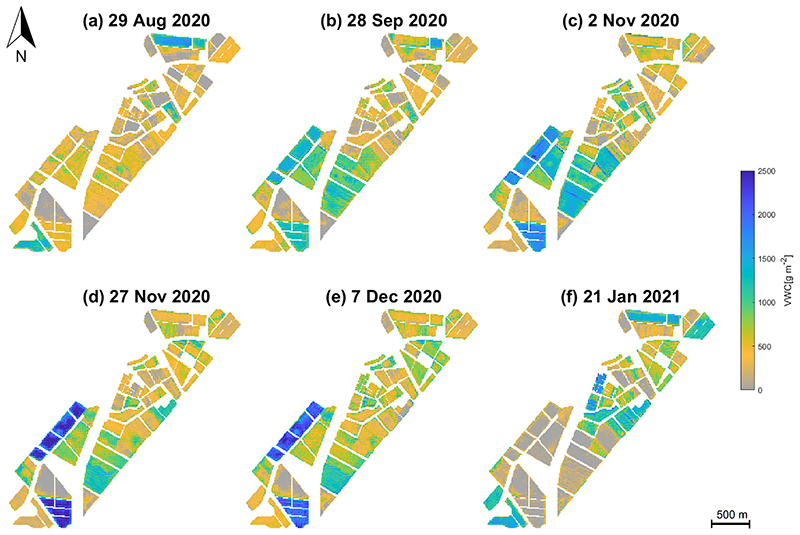
VWC [g m^−2^] of different crops in the study site, retrieved by the GPR model using simulated vegetation reflectance spectra, in-situ biophysical/biochemical measurements and S2 surface multi-spectral reflectance data. Data obtained from BVCR wheat campaign 2020. (**a**) wheat tillering stage at 29 August 2020; (**b**) wheat booting stage at 28 September 2020; (**c**) wheat anthesis-flowering stage at 2 November 2020; (**d**) wheat dough development stage at 27 November 2020; (**e**) wheat ripening stage at 7 December 2020; (**f**) harvested wheat at 21 January 2021.

VWC maps are displayed in Figure 9 for the six selected dates of the 2020 BVCR wheat campaign. These maps show spatio-temporal changes in VWC for all crop parcels at the BVCR study site, which were in parity with expected changes in VWC, as driven by rainfall conditions and irrigation management operations during the whole wheat’s phenological cycle. Since the VWC was calculated with FVC (see Equation (20)), VWC strongly depends on the percentage of bare soil present in the scene’s pixel observed by S2. FVC values widely vary during the vegetation growing period determined primarily by the crop development and structure as well as the implemented crop management practices. Typical VWC values for wheat crop vary from close to 0 g m^−2^ at the start and end of the season to around 2500 g m^−2^ in late November 2020.

### 3.3 Wheat Phenology Based on Multi-Temporal LAI Maps

According to the Z1.3 state of the Zadoks’ scale and collected field data, three leaves of wheat plants emerged in August 2020. Hence, most of the wheat pixels in the S2-observed scene have LAI < 0.5 m^2^ m^−2^. This is clearly noticeable in the LAI map produced for the date 29 August 2020 (see Figure 7a and Table 4). Later in the growing season, around 28 September 2020 (Z4.3, wheat at the tillering stage), all crop parcels revealed distinct LAI growing curves due to field irrigation and fertilization which took place by the third week of September. The S2-derived LAI map for the date 28 September 2020 reveals LAI values of about 2.5 m^2^ m^−2^ for the wheat crop (see Figure 7b).

Wheat plants were at the flowering stage on November 2 (Z6.1—few anthers at the middle of the ear) and the plants reached a height of around 80 cm. Hence, LAI ranged from 3.5 to 4.5 m^2^ m^−2^ by this date and almost all the cropped pixels at each paddock have experienced the beginning of the anthesis, resulting in higher LAI values (see Figure 7c). Note that some distinct heterogeneities are visible principally in paddock 322; it had a variety of vegetation vigor. In the anthesis stage, the wheat ear arises from the plant stalk, implying that all wheat paddocks are in the flowering stage.

The milk development stage started once the flowering is completed during the month of November, and wheat plants also reached their maximum greenness on 16 November 2020 (Z7.5—Medium milk). Moreover, senescence starts at this time and young leaves may become yellowish. Accordingly, wheat parcels had LAI > 4 m^2^ m^−2^, which represents the LAI curve’s inflection point hence from this date onwards LAI decreases until wheat harvest. The wheat traits maps show the early kernel formation stage which occurs one to two weeks after pollination. Once the plants’ dehydration period has begun the dough development stage starts. On 30 November (Z8.7—Hard dough), most of the kernel dry weight (starch and protein content) accumulates and therefore LAI < 3.5 m^2^ m^−2^ on average, this stage is also known as physiological maturity. The LAI map generated for the date 27 November 2020 displays the decrease of this biophysical variable over the BVCR study site (see Figure 7d) indicating the end of plant growth.

Finally, by the middle of December 2020, wheat parcels revealed complete senescence, and the seed moisture decreased down to 13 to 14%, which corresponds to the ripening stage. The wheat seeds could have been harvested at the end of this stage on 16 December 2020; however, wheat plants remained standing until the first days of January 2021. This can be appreciated in the LAI maps produced for the date 7 December 2020 where (green) LAI values oscillate between 1.5 and 2 m^2^ m^−2^ (see Figure 7e). At the end of the ripening stage, the seeds were harvested, which resulted in pronounced decrease in LAI on 4 January 2021 (see Figure 7f).

### 3.4 Seasonal Analysis of Retrieved Traits

**Figure 10:**
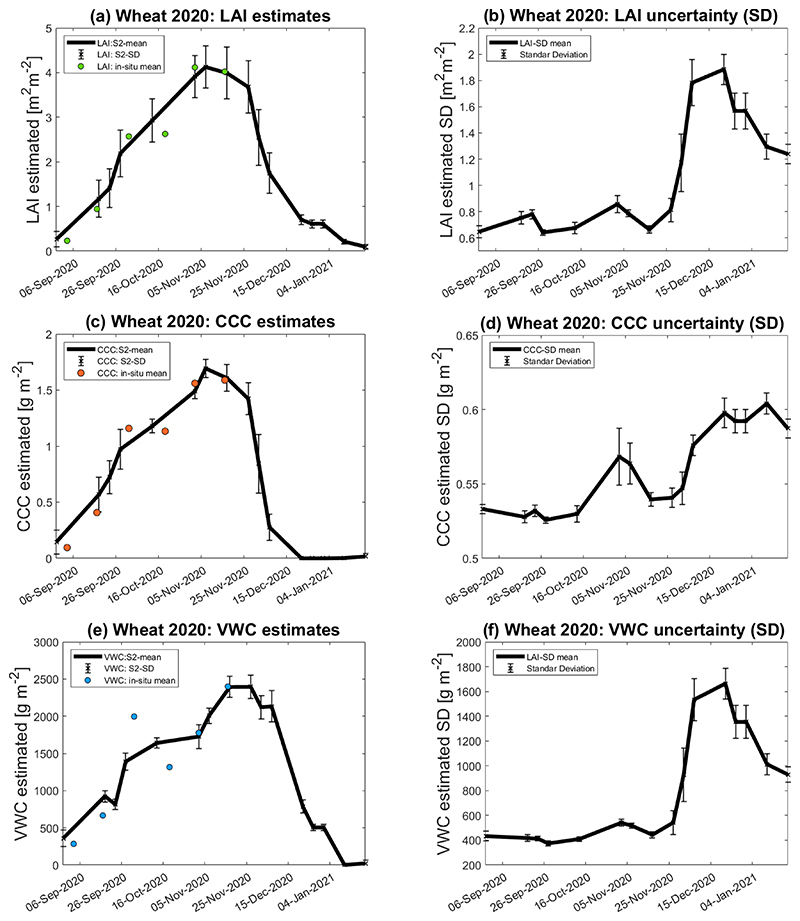
Seasonal evolution of wheat cropland over the three paddocks at the BVCR study sites described by LAI, CCC, and VWC mean values of nice ESUs within the crop limits and the associated uncertainty, plotted as vertical bars. (**a**) LAI estimates; (**b**) LAI uncertainty (SD); (**c**) CCC estimates; (**d**) CCC uncertainty (SD); (**e**) VWC estimates; (**f**) VWC uncertainty (SD).

This section explores the seasonal evolution of LAI, CCC and VWC along the wheat 2020 campaign. The trait models were applied to a total of 15 free-cloud L2A S2 scenes to obtain sufficiently dense time series along the wheat seasonal cycle. We also explored the GPR model uncertainty during the course of the season. The uncertainties are mapped in the form of SD to describe the variation of LAI, CCC and VWC values. LAI, CCC and VWC temporal gaps correspond to S2 cloudy acquisitions. Figure 10a displays the temporal profile of retrieved LAI and Figure 10b the LAI SD profiles of the nine in-situ measurement points corresponding to the three paddocks of wheat cropland in the BVCR study site. The figure shows the temporal evolution of the averaged nine ESUs (solid line), and the mean of the in-situ measured LAI values (yellow dots). Figure 10c,d show the temporal profile of the retrieved CCC, the SD, and the mean of the in-situ measured values of all ESUs. Figure 10e,f illustrate the VWC and SD trends. Seasonal patterns of LAI, CCC and VWC reproduce the typical phenological stages of the wheat crop. LAI, CCC and VWC increase steadily, reaching a maximum peak in November 2020. By that time, a plant’s dehydration process also emerges, and senescence starts. During the senescence stage, plant water content drops significantly, leading to the wilting of leaves and thus causing LAI to decrease [86]. Abrupt drops of LAI, CCC and VWC in January 2021 suggest the harvest event.

#### 3.4.1 In-Situ Measured FVC Time Series Analysis of Irrigated Winter Wheat Crops

**Figure 11:**
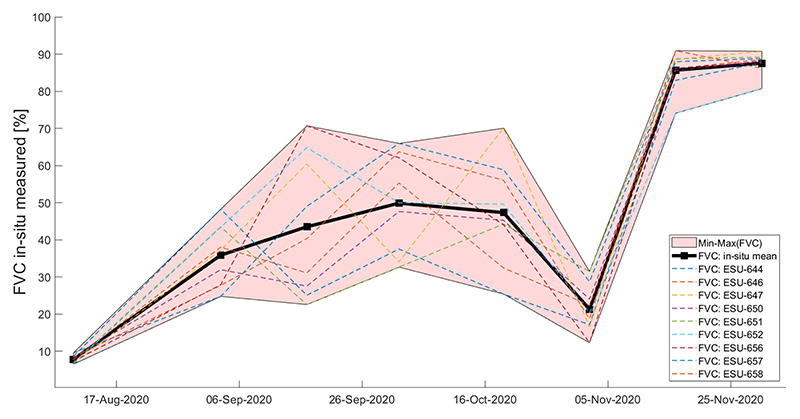
Temporal evolution of wheat crop over the three paddocks at the BVCR study sites described by FVC mean measured values of nine ESUs within the crop limits and the associated SD, which is plotted as shadowed areas.

**Figure 12:**
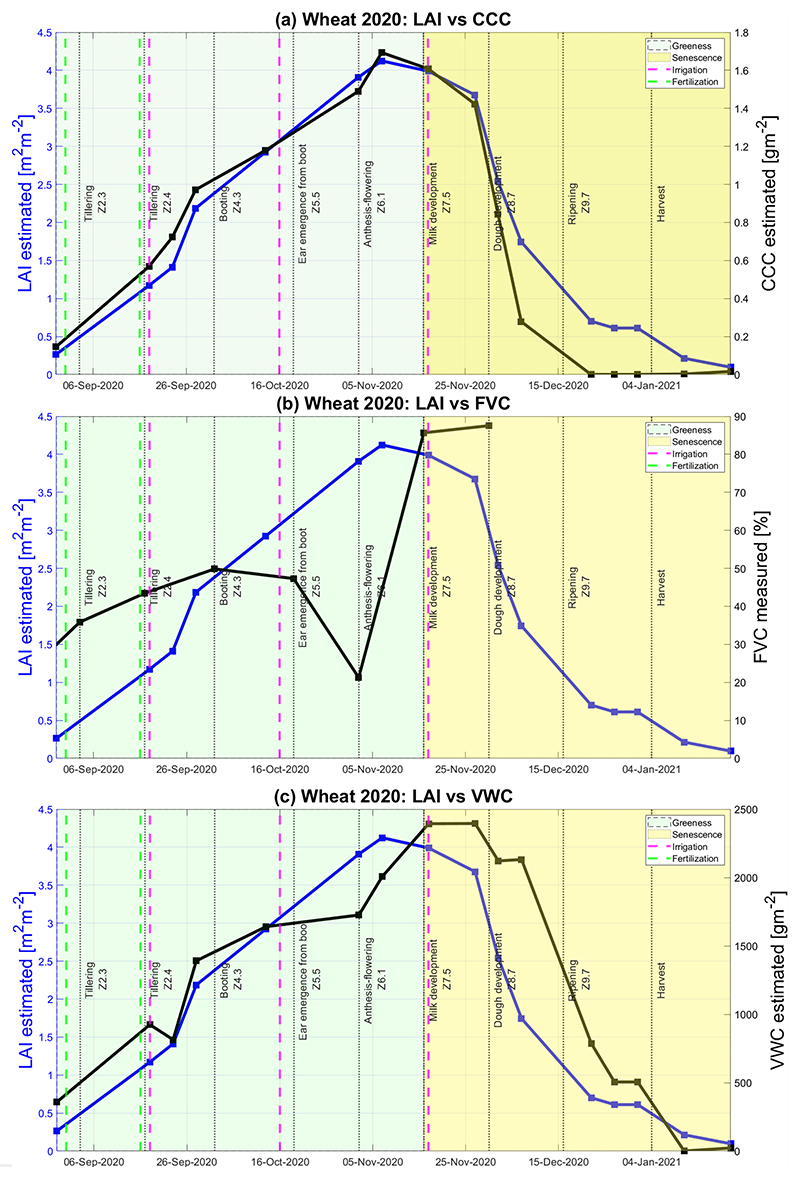
Temporal evolution of wheat cropland over the three paddocks at the BVCR study sites described by LAI, CCC and VWC mean values of nine ESUs within the crop limits and the associated SD, which is plotted as vertical bars. (**a**) LAI vs. CCC temporal estimates; (**b**) LAI temporal estimates vs. FVC in-situ measured values; (**c**) LAI vs. VWC temporal estimates.

In the course of wheat tillering, plants reach 10–20 cm height on 17 September and FVC values oscillate between 25–45% (see Figures 3c and 11). The crop leaves remain horizontal during the tillering stage with four leaves per tiller leading to increase of vegetation coverage to FVC about 30–65% on October 2 (see Figure 3d). From the booting stage onwards, the wheat FVC decreases due to plants’ stem elongation up to a minimum of 22% on average on November 2. Figure 12b, showing the seasonal patterns of the retrieved LAI vs. the in-situ measured FVC, reflects this behavior: a strong dropping of FVC at the beginning of November (minimum on 2 November), while LAI values remain high in this period (around 3.7 m^2^ m^−2^) and only decrease at the end of November with starting senescence. This specific behavior of FVC can be explained by the fact that the Canopeo®application uses only the RGB spectral bands to determine the crop canopy coverage.

Later on in the growing season, by November 16, the FVC values increase up to 86% (see Figure 11). During the senescence period, the FVC values remain high; wheat plants remain standing, and the FVC in-situ measured values range from 80 to 90.8% (see Table 2).

#### 3.4.2 Seasonal Analysis of S2-Retrieved CCC and LAI

Figure 12a presents the temporal profiles of the mean estimated CCC and LAI values along the wheat’s seasonal cycle. A strong positive correlation between LAI and CCC temporal profiles can be seen as C_*ab*_ values have been upscaled with LAI to obtain CCC. CCC retrieved values along the seasonal cycle of winter wheat range from 0 to 1.7 g m^−2^ approximately. At the start of the crop growing season and during the greenness phase, the CCC curve follows the LAI’s trend until 27 November. After the vegetation has reached the maximum greenness, which typically corresponds to the LAI peak value, the senescence phase begins until crops are ultimately harvested throughout January 2021. From 27 November onwards, during cropland senescence, the difference between LAI and CCC profiles starts to become more noticeable. On 7 December, 1.5 < LAI <2 m^2^ m^−2^indicates the presence of vegetation structures, while 0.2 < CCC < 0.4 g m^−2^ suggests that the chlorophyll content of wheat plants is declining until, finally, the crop turns yellowish due to the absence of the pigments. According to the in-situ collected notes (see Table 4), the complete senescence for the BVCR 2020 campaign of winter wheat crop at the study site took place by 16 December. Figure 12a shows how the CCC curve drops to zero from 22 December until 21 January. The agreement between both data sources confirms the capability of S2 to retrieve the CCC for monitoring winter wheat croplands.

#### 3.4.3 Seasonal Analysis of S2-Retrieved VWC and LAI

Figure 12c shows the seasonal evolution of the mean estimated value of VWC against LAI. As the VWC model is correlated to FVC in-situ measured values instead of LAI ones, a notorious time shift can be appreciated between the two curves. While the LAI curve describes the leaves’ surface per square meter of soil, VWC involves not only the leaves but also the stem and spikes of wheat plants. In addition, third wheat irrigation occurred on 17 November to boost the grain development. Consequently, although senescence had started on 16 November, the VWC values remain high at around 2300 to 2400 g m^−2^ until 27 November. Once the wheat plants have reached the complete senescence by the middle of December 2020, the mature stems remain with a certain level of humidity, thus VWC = 750 g m^−2^ approximately on 22 December.

## 4 Discussion

With the ambition to develop retrieval models for mapping the diversity of Argentinian winter wheat traits, time-series of S2 imagery was processed to obtain LAI, CCC and VWC maps along the phenological crop cycle in an irrigated, intensively cultivated area. This research is built upon a hybrid retrieval workflow using an optimised simulated training dataset and in-situ measurements of vegetation traits from the BVCR 2020 campaign. In the following, advantages and limitations of the RTMs (Section 4.1), performance of GPR models (Section 4.2), potential of the temporal vegetation traits mapping for wheat agronomic management (Section 4.3), study limitations (Section 4.4), and finally operational monitoring opportunities (Section 4.5) are discussed.

### 4.1 Advantages and Limitations of Coupled RTMs

Physically-based models simulating the influence of water content and pigments on the obtained signal acquired by satellite sensors potentially lead to improved predictions of crop traits compared to empirical approaches. Regarding the leaf level, we chose the PROSPECT model (i.e., the actual PROSPECT-PRO version), being perhaps the most widely applied leaf RTM in EO vegetation studies. PROSPECT had been first introduced in the 1990s by Jacquemoud et al. [87] and was continuously updated, with for instance improved calibration of the specific absorption coefficients of C_*ab*_ (introduced in PROSPECT-D [88]) and separation of dry matter content into leaf protein content and carbon-based constituents in PROSPECT-PRO [44]. Although results may not change significantly, the usage of the most actual model version is always recommended. Still, improvements to the model may be required, such as the inclusion of specular reflection [88].

To upscale from leaf to canopy level, C*_ab_* and EWT were multiplied by LAI simulated with the SAIL model to obtain CCC and VWC, respectively. The SAIL model assumes that the horizontal direction of the canopy is infinite [45]. Therefore, it is mainly suitable for estimating LAI and other traits of rather continuous and homogeneous crops [89]. Winter wheat is not a pronounced row crop (like maize) but belongs to the grass family (Poaceae). Hence, with its turbid medium assumptions, the choice of SAIL (or PROSAIL when combined with PROSPECT) is justified for our analysis. In addition, we used the simulated FVC data for driving calculations of the VWC training dataset (see Equation (1)). This approach differs from previous studies, e.g., [90,91] where canopy water content was obtained by upscaling EWT by LAI (see Equation (18)).

To optimise the simulated training dataset, we extensively explored the parameterization of winter wheat. Multiple settings were tested to achieve the final VWC model minimising the RMSE. The EWT values ranged from 0.0001 to 0.182 g cm^−2^, being the most convenient interval presented in Table 1. The in-situ collected variables, LAI, AGFB and AGDB, allowed us to calculate the EWT for winter wheat, involving plant leaves, stalks, and fruits. The calculated EWT values from the campaign data oscillated between 0.019 to 0.175 g cm^−2^, leading to an uncertainty increase in the final built model (RMSE = 587 g m^−2^). This can be argued by the fact that EWT implies only water content at the leaf level, whereas field measured data include the water content of all plant organs. Varying the EWT in the PROSAIL-PRO training dataset generation not only affected the VWC model statistics but also the R^2^ and RMSE of the LAI and CCC obtained models, which highlighted the sensitivity of PROSAIL-PRO to this leaf variable.

### 4.2 Performance of Hybrid GPR Models

The proposed workflow is built on the following fundamental factors. First, the careful design of the in-situ measurements methodology led to a collection of high-quality field wheat traits data. Second, the in-situ database covered multi-temporal vegetative stages. In this respect, the GPR models achieved high accuracy for the studied traits, with trustful estimates along the crop greenness phase. Third, we added non-vegetated spectra to the training dataset, i.e., from bare soils, man-made surfaces, and senescent crops as the retrieval models needed to be adapted to the spectral variability of actual land covers of the scenes. Note that, with these additional spectra in the learning process, a slight decrease in the validation may occur; however, more generic wheat traits models can be obtained [58,72].

Active learning provides a powerful and smart sampling method to decrease the redundancy of the training dataset maintaining the spectral variability and thus information content. Finally, from a total of 1000, 112 samples of LAI, 137 samples for CCC, and 232 samples for VWC were intelligently chosen to train the retrieval algorithms. In general terms, the three traits GPR models performed well in learning the nonlinear relationship between the reflectance of S2 spectral bands and LAI, CCC and VWC. This confirms the results of other hybrid studies [59,60,62], where these traits were successfully estimated from S2 data with a GPR model.

The provision of per-pixel associated uncertainty information helps to understand how trustful results are, required to appropriately use the information obtained through a model or measurement [92]. Such information can further indicate the portability of the methods in space and time and is important when the models are being tested across different crops or sites.

The seasonal analysis of LAI, CCC, and VWC revealed differences in the uncertainty for both green and senescent development phases of winter wheat. Generally, low uncertainties refer to spectra that were well represented during the learning phase, whereas retrievals with high uncertainties refer to spectral information that deviates from what has been represented during the training stage of the models [26]. During the growing season, the availability of in-situ measured traits renders the models more robust and confident. Additionally, the reasonably low uncertainty provided by the GPR retrieval models during this period for most paddocks at the study site suggests reliability in the mapping results. From 16 November onwards, the senescence started, and consequently, winter wheat spectra changed drastically. Additionally, field data were absent for this period; therefore, the uncertainty of the GPR models increased considerably (see Figure 10).

### 4.3 Potential of Seasonal Traits Mapping for Wheat Agronomic Management

Monitoring of winter wheat traits provides valuable information on the life cycle of the plants needed for efficient crop irrigation and fertilization management being critical factors to crop yield. Winter wheat in our study site was irrigated in the transition between phenological stages. Dates of fertilization were close to those of irrigation since the urea is incorporated into the soil with water, and fertilization occurs first followed by irrigation (see Figure 12). Wheat water absorption depends on the previous hydric stress condition as well as plants’ phenological stage. Effects on the vegetation water content can be noticed up to 4 or 5 days after crop irrigation [93–95]. Nitrogen fertilization increases the crop water use efficiency (WUE) due to the higher concentration of nitrogen in plants, leading to improved radiation use efficiency. C_*ab*_ is positively correlated to photosynthetic activity [96]; consequently, C_*ab*_ is an indirect indicator of plants’ total nitrogen content [97]. Plant stress conditions may affect the photosynthetic process generating nitrogen assimilation and carbon fixation reduction. The leaf chlorophyll content is a genetic attribute of wheat plants; consequently, it remains almost constant during most crop growth stages [3].

CCC monitoring is crucial to understanding plants’ photosynthetic activity. Repetitive LAI mapping of winter wheat can provide not only valuable information about plants’ leaf area but also a proxy for C_*ab*_ upscaling to canopy level. CCC monitoring provides useful information to infer plants’ salinity and water stress [98,99], as well for the early detection of nitrogen absorption deficiencies and decrease yield associated with plant stress conditions [14,100].

In Figure 12a, the winter wheat fertilization and irrigation dates are presented against the LAI and CCC trends throughout the crop season. The first and second irrigation take place during the tillering stage (Z2.3 and Z2.4 according to the Zadoks growth scale); as a consequence, the slope of the LAI and CCC curves increase. This analysis reveals the potential of the seasonal mapping of irrigated winter wheat traits in Argentina to manage the natural resource usage in arid and semi-arid regions.

The WUE of crops can only be roughly estimated, since, under field conditions, it is arduous to know precisely how much water has been consumed by plants and their growth in accumulated biomass [101]. Consequently, water consumption is usually estimated from indirect data on precipitation and the volume of water lost through runoff, percolation, or direct evaporation from the soil and which has never been consumed by plants [102,103].

Third irrigation occurs during the wheat’s milk development stage to boost grain yield (Z7.5 according to Table 4). Figure 12c shows the VWC curve response to this irrigation while LAI has started to decrease by the middle of November 2020.

Well-adjusted VWC models represent an easy-access tool for helping to obtain a response to the most fundamental questions: when and how much to irrigate winter wheat crops in arid and semi-arid regions. To support this analysis, one of the most tested and widespread tools is the hydric balance, which allows for estimating the water content in the soil and deciding the optimal moments of irrigation.

The data fusion of LAI, CCC, and VWC temporal profiles presented in our research constitutes a differentiating element in the field of remote sensing monitoring of irrigated winter wheat development at the study site. (see Section 3.4.2 and Section 3.4.3). The potential yield generated by irrigated wheat in the arid region of the focused study site can be compared with the grain production of the harvested winter wheat crops from the agricultural core area of Argentina. Consequently, monitoring crop traits results is crucial to reducing fertilizer and irrigation water uptake assuring high yields. All these statements point toward the potential of the vegetation traits retrieval models to address the agronomic analysis of irrigated winter wheat crops.

### 4.4 Study Limitations

Even though the in-situ collected data process was conscientiously carried out, the uncertainty of the measurements and used instruments were not considered in the analysis. However, uncertainty coming from field data has to be kept in mind when assessing the overall accuracy of the retrieval models.

From early August to mid-November, winter wheat plants remain green; thereby, the in-situ LAI samples correspond to the crop’s growing season. During this period green, LAI (i.e., GAI) increases as the wheat plants grow. Once crop senescence arrives, plants remain standing and the share of brown leaves increases [13,68]. Each of the winter wheat paddocks was revisited periodically from 10 August to 16 December 2020. The in-situ LAI values were taken from 3 September to the end of the BVCR 2020 wheat campaign; nevertheless, only the green LAI samples were considered to train the LAI model (from 3 September to 16 November). From 16 November to 16 December, in-situ LAI values continue to rise although the cropland has become senescent (see Table 2).

Since in-situ LAI values were used to tune the GPR retrieval models with the AL-EBD techniques, the final LAI retrieval model is better adapted toward green vegetation states. Hence, the uncertainty of the developed models will increase during later (mature/senescent) growing stages.

Finally, it must be noted that our study was conducted with data coming from one single growing season. Thus, temporal transferability to other seasons remains an open question. However, since GPR models provide associated uncertainty estimates, the fidelity of temporal and spatial transferability of the retrieval models to other regions with different cropping environments can be assessed to some degree [104].

### 4.5 Opportunities for Operational Monitoring

The possibility to estimate the irrigated winter wheat traits at 10 m or 20 m GSD would offer important opportunities for operational precision farming applications. The promising line of hybrid retrieval workflows opens some new branches to be explored in the field of agricultural applications using EO data [105]. The following questions arise: (1) Can we extrapolate the traits models for winter wheat crops to other regions and times? (2) Are the crop traits models sufficiently generic to be applied to other crop types in arid and semi-arid intensively cultivated areas? (3) Are the outcomes of this research useful to address the management of the crops more efficiently, for instance determining when to irrigate and to fertilize optimally?

In this context, the availability of the winter wheat in-situ database obtained from the BVCR 2021 campaign represents an excellent opportunity for the validation and refinement of the developed models and will be explored in a future study.

With respect to the implementation of the GPR models into operational processing, recent possibilities are opened thanks to cloud computing technologies. In a pioneering study, [106] presented an approach on how to integrate GPR models into Google Earth Engine (GEE), e.g., for S2 processing. In this way, the GPR models can be applied anywhere and anytime in the world. Pursuing this approach, first applications already emerged regarding multiple crop traits mapping and monitoring of key growing cycle events (e.g., start/end-of-season) [60] or for wide-scale S2 cropland traits mapping, e.g., over the entirety of Germany [62]. GPR models developed here can be likewise implemented into GEE for monitoring applications. For instance, such a straightforward monitoring interface can help users to understand the appropriate rates and timing of fertilization and irrigation.

## 5 Conclusions

We presented a hybrid retrieval workflow for operational mapping of LAI, CCC, and VWC that was optimised for an irrigated winter wheat cropland.

The implemented hybrid retrieval method used the advantages of the radiation-surface physics interaction being considered by the RTM and at the same time flexibility, scalability, and computational speed that are provided by GPR. The usage of GPR as a solid probabilistic regression algorithm provides the additional advantage of delivering associated uncertainties along with the trait estimates, helping to assess the fidelity of the mapping results.

The hybrid models for the winter wheat traits were validated with relative high accuracies against in-situ data (LAI: RMSE = 0.43 m^2^ m^−2^; CCC: RMSE = 0.27 g m^−2^; VWC: RMSE = 416 g m^−2^). They were subsequently applied to a series S2 imagery during the growing season of 2020 over the South of Buenos Aires Province, Argentina. All produced winter wheat maps showed consistent spatiotemporal performance and relatively low associated uncertainty intervals. The seasonal evolution of the winter wheat crops was quantified by correlating in-situ collected data and interpreting crop growth stages of the S2-derived maps. We conclude that routine mapping of wheat LAI, CCC and VWC constitutes a unique opportunity to monitor irrigated winter wheat development, which will contribute to managing sustainable agricultural production and thus assuring food security.

## Supplementary Material

Appendix A

## Figures and Tables

**Table 1:** Parameter ranges for PROSPECT-PRO and 4SAIL (PROSAIL-PRO). Specified ranges are uniformly distributed in LHS sampling.

Leaf Optical Properties			Canopy Reflectance Model		
PROSPECT-PRO Parameters	Notation [Unit]	Range	4SAIL Parameters	Notation [Unit]	Range
Leaf chlorophyll a+b content	*Cab* [μg cm^−2^]	5–75	Leaf area index	*LAI* [m^2^ m^−2^]	0.1–7.0
Leaf structure parameter	*Nstruct, no dim.*	1.0–2.0	Average leaf inclination angle	*ALIA* [°]	30–70
Leaf carotenoid content	*Cxc* [μg cm^−2^]	0–15	Soil brightness	*soil, no dim.*	0–1
Leaf equivalent water thickness	*EWT* [cm]	0.0002–0.05	Sun zenith angle	*SZA* [°]	20–40
Carbon-based constituents	*CBC* [g cm^−2^]	0.001–0.01	Hot spot effect	*Hot* [m m^-1^]	0.01
Leaf anthocyanin content	*Canth* [μg cm^−2^]	0–2	Observer zenith angle	*OZA* [°]	0
Leaf protein content	*Cp* [μgcm^−2^]	0.001–0.0025	Diffuse/direct radiation	*DDR* [%]	80
Leaf mass per area	*Cm* [μg cm^−2^]	0.0001–0.03	Relative azimuth angle	*rAA* [°]	0
Brown pigment content	*Cbrown, no dim.*	0			

**Table 2:** Database of in-situ wheat traits with dates, ranges, mean values as well as standard deviations (SD) of the measurements.

Wheat Variable	Date	Range	Mean	SD
**LAI** (m^2^ m^−2^)	03-09-2020	0.16–0.30	0.23	0.05
	17-09-2020	0.56–1.54	0.94	0.29
	02-10-2020	1.59–3.81	2.57	0.66
	19-10-2020	1.53–3.27	2.62	0.51
	02-11-2020	2.78–5.05	4.12	0.63
	16-11-2020	3.31–5.39	4.02	0.80
	30-11-2020	3.29–4.75	4.08	0.50
	16-12-2020	3.97–5.64	4.68	0.43
**FVC** (%)	10-08-2020	6.2–9.1	7.63	1.00
	03-09-2020	23.0–48.0	34.94	8.32
	17-09-2020	22.1–80.2	44.21	22.73
	02-10-2020	32.7–69.2	48.30	13.28
	19-10-2020	23.6–69.5	46.15	12.97
	02-11-2020	11.1–29.3	20.92	5.07
	16-11-2020	74.4–92.0	87.44	4.83
	30-11-2020	80.0–90.8	88.22	3.06
C_*ab*_ (μg cm^−2^)	03-09-2020	38.23–44.82	41.96	2.23
	17-09-2020	36.49–52.61	42.21	4.91
	02-10-2020	38.12–52.02	45.33	4.46
	19-10-2020	39.64–45.12	43.08	1.84
	02-11-2020	33.63–42.44	38.29	2.89
	16-11-2020	35.72–44.92	39.41	2.85
	30-11-2020	13.93–48.31	35.32	9.59
**AGFB** (g)	03-09-2020	15–25	19.67	3.59
	17-09-2020	31–54	42.00	6.83
	02-10-2020	76–175	112.67	27.23
	19-10-2020	47–94	66.00	13.61
	02-11-2020	131–296	213.67	42.41
	16-11-2020	57–101	81.78	15.84
	30-11-2020	73–184	121.60	38.45
**AGDB** (g)	03-09-2020	2.00–6.00	3.56	1.17
	17-09-2020	9.00–15.00	11.67	2.00
	02-10-2020	23.00–48.00	33.22	8.23
	19-10-2020	8.00–15.00	12.11	2.42
	02-11-2020	38–62	45.67	7.86
	16-11-2020	17–32	26.00	4.22
	30-11-2020	23–70	46.67	14.04

**Table 3:** Dates, ranges, mean values, and standard deviations (SD) for calculated variables (CCC and VWC).

Wheat Variable	Date	Range	Mean	SD
**CCC** (g m^−2^)	03-09-2020	0.06–0.12	0.10	0.02
	17-09-2020	0.22–0.71	0.41	0.16
	02-10-2020	0.74–1.61	1.16	0.29
	19-10-2020	0.60–1.44	1.13	0.23
	02-11-2020	1.18–1.74	1.56	0.17
	16-11-2020	1.22–2.10	1.59	0.34
	30-11-2020	0.64–1.70	1.40	0.29
**VWC** (g m^−2^)	03-09-2020	207–455	284	70
	17-09-2020	315–1554	666	360
	02-10-2020	868–3021	1996	693
	19-10-2020	494–2240	1315	554
	02-11-2020	944–3083	1777	629
	16-11-2020	1481–3275	2399	589
	30-11-2020	2016–5079	3287	1113

**Table 4:** Winter wheat field campaign observations with in-situ sampling dates, crop growth stages and details of the field observations.

In-Situ Measurements Date	Wheat Growth Stage	Field Observations
10-08-2020	Seedling growth Z1.3—Three leaves emerged	Plant density: 248 plants m^−2^ (on average), previous crop: sunflower for seed
03-09-2020	Tillering, 2–3 tillers, Z2.3—Main stem and three tillers	Leaves per tiller 2 + 1 flag leaf
17-09-2020	Tillering, 4 tillers Z2.4—Main stem and four tillers	Leaves per tiller: 3 Irrigation date: 17/09/2020 Fertilization date: 16/09/2020
02-10-2020	Tillering, 5 tillers 4—Booting Z4.3—Boots just visible swollen	Plants height 22 cm from the base to the second node Leaves per tiller: 4
19-10-2020	Ear emergence from boot Z5.5—Ear half emerged	Plants height 71 cm (on average) Plants stem nodes: 5
02-11-2020	Anthesis (flowering) Z6.1—Beginning of anthesis (few anthers at the middle of ear)	Plants height 80 cm (on average)
16-11-2020	Milk development Z7.5—Medium milk	Plants height 80 cm (on average) Start of the senescence
30-11-2020	Dough development Z8.7—Hard dough	Senescence process
16-12-2020	Ripening Z9.7—Seed not dormant	Complete senescence Distance between rows: 19 cm Number of ears at 0.50 cm: 72 on average

**Table 5:** Field campaign and Sentinel-2 acquisition dates.

In-Situ Measurements Date	S2 Acquisition	±Δ Days
10-08-2020	NA	NA
03-09-2020	29-08-2020	–5
17-09-2020	18-09-2020	+1
02-10-2020	28-09-2020	–4
19-10-2020	13-10-2020	–6
02-11-2020	02-11-2020	0
16-11-2020	17-11-2020	+1
30-11-2020	NA	NA
16-12-2020	22-12-2020	+6

## Data Availability

Not applicable.
