# Supplementary material for: Seasonal Mapping of Irrigated Winter Wheat Traits in Argentina with a Hybrid Retrieval Workflow Using Sentinel-2 Imagery
Source: Remote Sens (Basel). Author manuscript; Available in PMC 2022 Sep 29. (PMC7613660; doi:10.3390/rs14184531)
Supplement: Appendix A [file EMS154472-supplement-Appendix_A.pdf]

implies a sufficient degree of generalization to ensure that the model is applicable to S2 time series data and able to retrieve the vegetation traits across the complete phenological cycle.

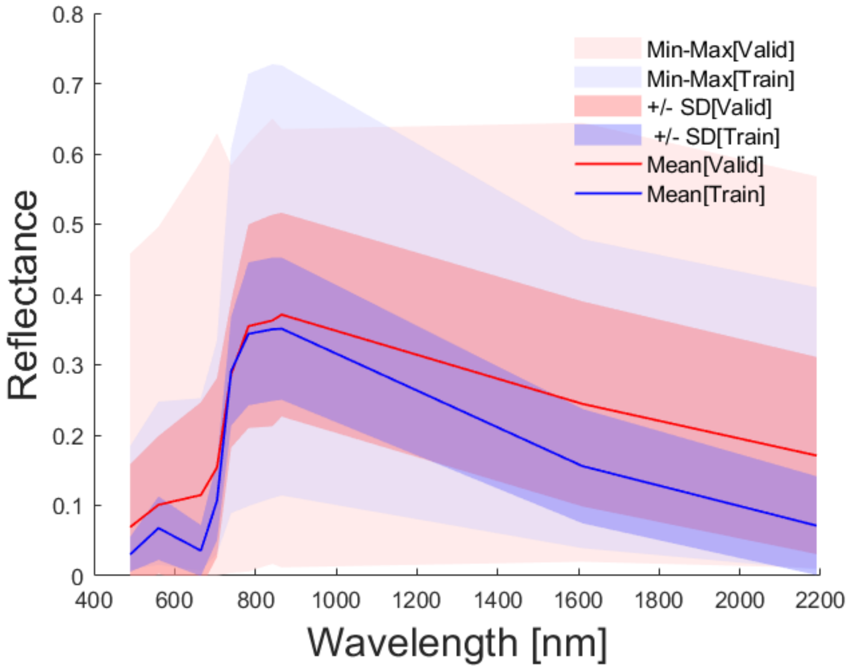

Figure A1. Statistics(mean, standard deviation, min–max) of EBD-reduced final training dataset (blue) vs. validation dataset (red). Training data base was simulated with PROSAIL-PRO.).
